# The Glorious Fourth and the Inglorious Fifth

**Published:** 1904-06

**Authors:** 


### Abstracts and Selections.

### The Glorious Fourth and the Inglorious Fifth.

After the smoke and noise of the last celebration had died away and the returns of the annual holocaust were in, *The Journal* summed up the cost of life and limb as it had been done before, and published the totals with their full and dreadful significance. Since that time we have had many reasons for being glad that we did the work and presented its results, for throughout the country many earnest workers for civic betterment have availed themselves of our figures to use as argument to secure the passage of ordinances directed to the prevention of these mournful features of a day of noise and turmoil. From many States have come newspaper accounts of city councils passing bills directed against the sale of toy pistols, blank cartridges and giant crackers to minors, and quoting the figures that we published last August—106 deaths from lockjaw, 60 from other causes, and 3,893 injuries that were not fatal.

The time has now come to look forward once more to the coming Fourth, and to see if, in the light of our past experience, a complete repetition of the annual calamity can not be avoided. It is evident that a certain amount of public sentiment has been stirred up in one way and another, but whether it will assume its activity at the proper time is another question—the most general condemnation of the method of celebrating the Fourth, and the loudest calls for a change are usually heard about August 1. In Chicago last year a pathetic example of this was furnished. There have been standing on the city statutes of Chicago for many years ordinances providing penalties for the sale of pistols to minors, but their enforcement has never been anything worth mentioning. In the latter part of June, 1903, after a few premature lockjaw cases had followed blank cartridge injuries, some desultory arrests of venders of these weapons were made, but no general effort was made to close down on this source of trouble. The small boy had not the slightest difficulty in procuring the materials for his destruction, as we easily demonstrated by experiment. But when the daily papers began to fill with reports of deaths from lockjaw a few days after the Fourth, *then*, and not until then, was any determined effort made to stop the sale of toy pistols. At that time we found that a boy in knee trousers had to go sometimes to as many as two or even three stores before he could purchase a toy pistol.

The lesson is plain. Something must be done now to awaken the police departments of our cities to their duties in this direction. Last year 64 per cent of the total injuries and over 80 per cent of the fatal injuries were the results of blank cartridges. Nearly all cities, it would seem, have at some time passed ordinances that control their sale, or the sale of the pistols in which they are used, but, as a rule, the ordinances have remained on the books unconsulted and non-enforced. It would not be a difficult matter to reduce this source of mortality greatly; the pistols and cartridges are sold usually by dealers in sporting goods or in "notion stores," and every policeman knows all such places on his beat. Confiscation of pistols from boys on the Fourth would also be of some service. Enforcement of the law is the chief thing that is needed. In Washington, where these matters seem to be controlled better than in ordinary municipalities Fourth of July tetanus is extremely uncommon, although there are a fair number during the year from other causes.

It seems to us that there is a good field for the activities of local medical societies in this particular branch of preventive medicine. Passing of resolutions requesting the enforcement of such laws, if they exist, or the drafting of them where lacking, if sent from representative bodies of medical men to the proper authorities, and if published in the daily papers to create popular interest, would not fail to be of effect either immediately or remotely. It will be found that, with few exceptions, the daily papers are ready and eager to take up this cause, and they furnish powerful aid. Last year much good work was done by the press.

A good suggestion is to be found for other cities in the establishment in Chicago of an incorporated association—the Chicago Amusement Association—with the purpose of improving Fourth of July celebrations. It is planned by this organization, which is supported by popular subscription, to secure enforcement of the laws, to aid in prosecution when they are violated, and particularly to do everything possible to educate the public as to the necessities of the situation.—*Journal American Medical Association.*

